# Esperience in confronting the H1N1 epidemy

**DOI:** 10.1186/1753-6561-5-S6-P85

**Published:** 2011-06-29

**Authors:** ACDSD Oliveira, S Scota, AM Costa E Silva, ESD Abreu

**Affiliations:** 1 Educação Continuada, Instituto de Infectologia Emílio Ribas, São Paulo, Brazil

## Introduction / objectives

The epidemic influenza A (H1N1) virus is the result of reassortment between genetic material from the human, avian and swine virus. In July 2009 the World Health Organization published the sustained transmission of the virus in two continents, characterizing the epidemy and occurring prompt response by this agency worldwide. The objetive to report the experience of the multidisciplinary team regarding adherence to and effectiveness of this training.

## Methods

The survey was conducted in a public hospital of Infectious Diseases, Brazil. Hospital Infection Control, Nursing Continuous Education and medical staff assembled to determine the preventive actions and the routines that would be adopted to confront the epidemy. These actions were based on Standard Precautions and guidelines of the Ministry of Health, Centers for Disease Control (CDC) and the Epidemiological Surveillance Center of São Paulo. Training was carried out from 08 to 21 May, 2009 covering the entire hospital staff, broaching precautions and isolation, management of suspected and confirmed cases and hospitalized patients.

## Results

357 employees were trained, with good adhesion, improved adherence to preventive practices and routines and substantial increase in the use of alcohol gel by institution professional staff. There was a low rate of retirement of-hospital employees due to infection by the virus. The difficulties encountered during training were due to the constant change in the guidelines issued by the official bodies by dealing with a relatively new disease.

## Conclusion

Improvement in hospital infection control measures such as hand hygiene, importance of teamwork, as well as permanent education as strategies to implement effective measures for providing quality care.

## Disclosure of interest

None declared.

